# Residual XGBoost regression—Based individual moving range control chart for Gross Domestic Product growth monitoring

**DOI:** 10.1371/journal.pone.0321660

**Published:** 2025-05-09

**Authors:** Rahida Rihhadatul Aisy, Latifatuz Zulfa, Yolanda Rahim, Muhammad Ahsan

**Affiliations:** Department of Statistics, Faculty of Science and Data Analytics, Institut Teknologi Sepuluh Nopember, Surabaya, Indonesia; Atlantic Technological University, IRELAND

## Abstract

Accurate and reliable Gross Domestic Product (GDP) forecasting is indispensable for informed economic policymaking and risk management. Autocorrelation, a prevalent characteristic of macroeconomic time series, poses significant challenges to traditional forecasting methodologies and statistical process control. This study introduces a novel approach to GDP forecasting and monitoring by integrating XGBoost regression, a robust machine learning algorithm, with Individual and Moving Range (I-MR) control charts. By effectively capturing complex nonlinear relationships and mitigating autocorrelation, the proposed model offers enhanced predictive accuracy compared to conventional methods. Empirical results demonstrate the model’s efficacy in phase I, aligning closely with actual GDP values. However, phase II analysis reveals discrepancies, suggesting the need for further model refinement and the potential incorporation of additional economic indicators to improve forecast precision.

## Introduction

Economic growth is quantitatively assessed by a nation’s GDP changes. GDP represents the aggregate monetary value of all final goods and services produced within a country’s geographic boundaries over a specific period, typically one year. This metric encapsulates the total remuneration accruing to the factors of production employed in the domestic production process [1]. GDP is a macroeconomic indicator that quantifies the total monetary value of final goods and services produced within a nation during a given period. Commonly used as a proxy for a country’s economic health and aggregate demand, GDP serves as a fundamental measure for assessing economic growth, productivity, and living standards. Moreover, GDP is a primary criterion for membership in the Group of Twenty (G20), an intergovernmental forum of the world’s largest economies. The G20’s focus on global economic stability, financial regulation, and sustainable development necessitates the inclusion of countries exhibiting significant GDP, indicative of their economic influence and capacity to contribute to these objectives [2]. The G20 serves as a key forum for global economic governance, bringing together the world’s twenty largest economies. Indonesia, a regional economic powerhouse in Southeast Asia, is a prominent member of this influential group, primarily due to its substantial population and economic scale. While other Southeast Asian nations, such as Malaysia, Thailand, Brunei Darussalam, and Singapore, also exhibit significant regional economic influence, Indonesia remains the sole representative of the region within this exclusive body [3].

GDP is a primary metric for assessing a nation’s economic activity and overall health. Accurate GDP forecasting is instrumental in informing effective policy formulation, investment strategies, and macroeconomic stabilization. The Autoregressive Integrated Moving Average (ARIMA) model has established itself as a prevalent time series methodology for modeling GDP fluctuations. Its efficacy stems from its capacity to capture the inherent autocorrelation and temporal patterns characteristic of economic data [4,5]. Huda et al. employed a Generalized Space-Time Autoregressive (GSTAR) model to examine the spatial and temporal determinants of GDP growth in Indonesia, Malaysia, Singapore, Thailand, and Brunei Darussalam. Their findings indicate that a uniform spatial weight matrix effectively captures the spatial dependencies among these economies, suggesting a homogeneous spatial structure in GDP growth within the region [6]. In recent years, Long Short-Term Memory (LSTM) neural networks have emerged as a promising tool for GDP forecasting. These models have demonstrated superior performance in capturing complex temporal dependencies within economic data, resulting in enhanced predictive accuracy compared to traditional time series methods [7,8].

To effectively monitor GDP trends, time series modeling can be employed in conjunction with control chart analysis. A fundamental assumption underlying control chart methodology is the independence and uncorrelated nature of observations. In the context of time series analysis, where data points exhibit inherent dependencies, residuals derived from an appropriately specified time series model are employed as control chart inputs. This approach is justified by the assumption that such residuals approximate white noise, characterized by independence and zero autocorrelation. Consequently, control charts can be effectively applied to any time series process for which a white noise residual structure is tenable. Time series models serve to mitigate the impact of autocorrelation, a statistical property that can obscure genuine process changes and lead to erroneous conclusions about GDP stability [9]. To monitor autocorrelated data, a modified control chart can be implemented by analyzing residuals derived from a time series model. As suggested by Yaschin, for data exhibiting a high degree of autocorrelation, a residual-based control chart is a suitable approach [10]. A study by Imro’ah et al. investigated the applicability of residual control charts for monitoring the forecasting accuracy of ARIMA models in the context of GDP prediction. They employed an Individual Moving Range control chart to analyze the residuals obtained from the fitted ARIMA model. The diagnostic tests revealed a lack of normality in the residuals, suggesting potential model misspecification. Furthermore, the I-MR control chart identified observations exceeding the control limits, indicating the presence of outliers or significant deviations from the predicted values. These findings collectively suggest that the chosen ARIMA model may not be sufficiently accurate in capturing the underlying dynamics of GDP and, consequently, may not be reliable for forecasting future values [11]. The I-MR chart is employed to graphically monitor individual data points collected sequentially. The I chart tracks the central tendency of the process, while the MR chart provides insights into process variability. By examining both charts concurrently, practitioners can identify potential shifts in the process mean or increases in dispersion [12,13].

Integrating control charts with machine learning models such as support vector machine (SVM), Neural Networks, and Xtreme Gradient Boosting model (XGBoost) offers various advantages and disadvantages for tracking GDP predictions. Control chart with SVM effectively monitor the stability of predictions over time and detect shift of trends in GDP values. Moreover, SVM’s computational efficiency can be a limitation when dealing with very large datasets, requiring further optimization techniques. Neural Networks, while highly accurate for capturing non-linear relationships, required significantly more computational resources and larger datasets for training to achieve optimal results. On the other hand, XGBoost known for its high performance and speed, can monitor prediction consistency, detect deviations in GDP trends, and offer robustness to missing data while providing feature importance insights.

To address the inherent limitations imposed by the autocorrelation assumption prevalent in prior research, this paper introduces a novel modeling framework utilizing XGBoost regression. One of the primary reasons for selecting XGBoost regression is its accuracy. The algorithm has consistenly outperformed other machine learning models. For GDP prediction, where precision is crucial, XGBoost’s ability to minimize prediction errors through techniques like regularization and cross-validation is invaluable [14]. Because of XGBoost’s effectiveness in managing huge datasets and capacity to reduce overfitting, it has been extensively utilized in the context of forecasting and classification [15]. The XGBoost model successfully modeled the dynamic behavior of data, accurately predicting the full range of values, including extreme events, and closely reflecting the observed flow patterns [16]. By explicitly acknowledging and accommodating the temporal dependencies inherent in the data, this approach seeks to enhance predictive accuracy and robustness compared to traditional methods that implicitly assume independence between observations. Given the extensive time range (1976–2021) and the inclusion of multiple countries, such as Indonesia, Brunei Darussalam, Malaysia, Singapore, and Thailand, the dataset is quite large. XGBoost’s scalability ensures that it can handle this extensive dataset efficiently, making it a suitable choice for the study. Kovarik et al. use control charts for time series analysis in financial management [17]. The researchers explain the use of control charts in case studies involving the analysis of Slovak currency and Argentina’s Gross Domestic Product highlights the flexibility of this method in managing cash flows and financial stability. Control charts such as CUSUM, EWMA, and ARIMA are not only capable of monitoring heteroskedastic financial processes but can also be applied in various economic contexts to detect small changes that might be overlooked by conventional methods [17]. Sulistiawanti et al. implemented a hybrid modeling approach to enhance water quality surveillance at multiple Water Treatment Plants (WTPs). The researchers leveraged Multivariate Exponentially Weighted Moving Average (MEWMA) and Multivariate Exponentially Weighted Moving Variance (MEWMV) control charts to monitor and detect anomalies in key water quality parameters. To address the inherent autocorrelation present in hydrological and water quality data, the study incorporated residual XGBoost regression as a preprocessing step, thereby improving the sensitivity and reliability of the control charting methodology in identifying process instabilities [18]. Therefore, the study that will be discussed in this research is the control of GDP growth from five countries as G20 members, using an I-MR control chart based on residual XGBoost regression.

Based on aforementioned reason, this research investigates the application of I-MR control charts to monitor and potentially regulate the GDP growth trajectories of five G20 nations. A novel approach is proposed, utilizing the residuals derived from XGBoost regression as the input data for the control charting process. This methodology aims to enhance the precision and sensitivity of identifying anomalies in GDP growth patterns, thereby providing early warning signals for potential economic instabilities. Detected anomalies or out-of-control signals in GDP growth can provide critical insights for policymakers and economists. These signals may indicate underlying economic issues such as inflation, unemployment, or supply chain disruptions. By identifying these anomalies early, policymakers can implement targeted interventions, such as adjusting interest rates, modifying fiscal policies, or introducing stimulus packages, to stabilize the economy. Economists can also use this information to forecast future economic trends and advise on long-term strategic planning.

## Materials and Methods

### Data source

This study employs secondary time-series data on GDP procured from the World Bank database. The sample encompasses five ASEAN nations: Indonesia, Brunei Darussalam, Malaysia, Singapore, and Thailand, spanning the period from 1976 to 2021, resulting in a total of 46 annual observations. To account for potential structural breaks in the GDP series, the data is partitioned into two phases. Phase I covers the period from 1976 to 2010, while Phase II spans 2011–2021. This delineation is informed by visual inspection of the time series plot which reveals discernible shifts in the GDP trajectories, likely attributable to the lingering effects of the 2008–2009 global financial crisis. The post-crisis period (Phase II) is characterized by the ASEAN economies’ efforts to restore economic equilibrium, consequently influencing the GDP growth dynamics within the region [19].

### Data analytical method

#### Xtreme Gradient Boosting Model.

XGboost is a machine learning ensemble method that employs gradient boosting of decision trees. By iteratively constructing a set of decision trees, each aiming to correct the errors of its predecessors, XGBoost produces highly accurate predictive models. Recent comparative studies have demonstrated that XGBoost consistently outperforms traditional empirical formulas and numerical models in terms of predictive performance and computational efficiency [20]. XGBoost has been used extensively and has produced excellent outcomes for a wide range of issues. Because of its features for regression, classification, and ranking, this version of the Gradient Boosting Method (GBM) is more effective and scalable than previous modelsXGBoost is also a widely implemented method in machine learning and data mining challenges, with the winning team’s solutions including XGBoost or ensemble XGBoost with neural networks. This system operates ten times quicker than the most widely used versions currently in use [15].

The objective function of the XGBoost model is expressed as follows [21]:

${\text{Y}}^{\text{k}}=\sum {\mathrm{\backslash nolimits}}_{\text{i}=1}^{\text{n}}\text{l}\left(\left({\text{y}}_{\text{i}},{\text{y}}_{\text{i}}^{\text{k}-1}\right)+{\text{f}}_{\text{k}}\left({\text{x}}_{\text{i}}\right)\right)+\;\;\;\Omega \;\;\;\left({\text{f}}_{\text{k}}\right)$ (1)

where n is the number of training data, ${\text{x}}_{\text{i}}$ represents the feature vector and ${\text{y}}_{\text{i}}$ represents the label on the 𝑖 -th instance, ${\text{y}}_{\text{i}}^{\text{k}-1}$ represents the prediction of the 𝑖 th instance at the t−i-th iteration, l is the loss function that measures the difference between the label and the final prediction plus the result of the new tree. ${\text{f}}_{\text{k}}$ represents the new tree that classifies the 𝑖 -th instance using ${\text{x}}_{\text{i}}$, and denote the regularization term that reduces the complexity of the new tree.

The primary objective of XGBoost is to continuously add weak trees with different weights to the ensemble of models. These trees should approximate the residuals of the previous predictions as closely as possible, which is expressed as follows [22]:

${\hat{\text{y}}}_{\text{i}}=\sum {\mathrm{\backslash nolimits}}_{\text{k}-1}^{\text{k}}{\text{f}}_{\text{k}}\left({\text{x}}_{\text{i}}\right)\;{\text{f}}_{\text{k}}\in \text{F}$ (2)

Where $\hat{\text{y}}$ where is the prediction result, and F represents the set that includes all regression trees, ${\text{f}}_{\text{k}}$ where represents one of the K regression trees, and *k* is the number of regression trees. The primary objective is to ensure that the predicted value ${\hat{\text{y}}}_{\text{i}}$ is as close as possible to the actual value ${\text{y}}_{\text{i}}$, While still maintaining generalizability. For many years, this technology has garnered significant attention due to its exceptional performance in terms of high efficiency and accuracy, ease of interpretation, flexibility, and scalability. For instance, it can handle large-scale data in parallel with high efficiency and can iteratively optimize the model, which generally results in better prediction accuracy compared to other algorithms [23].

### Individual-moving range control chart

The basic assumption in control charts is that observations must be independent and uncorrelated with each other. In a time series control chart, residuals are used as observations because, in time series models, residuals are assumed to be independent and uncorrelated white noise. Therefore, control charts can be applied to any time series model that assumes white noise in its residuals.

A control chart that meets this condition is the I-MR chart. In many situations, the sample size used for process monitoring is n =  1; that is, the sample comprises individual units. To supplement the controlling, a moving range chart supports successive absolute differences | X_i_ – X_i-1_ | as the basis of estimating the process variability [24]. Where the calculation can be written as follows [11]:

$\begin{array}{c} \text{Individual }\;\;\;\;\;\text{ plot }\;\;\;\;\;\;\left\{ \begin{array}{c} \text{UCL}=\bar{X}+3\frac{\overset{―}{\text{MR}}}{{\text{d}}_{2}}\\ \text{CL}=0\\ \text{LCL}=\bar{X}-3\frac{\overset{―}{\text{MR}}}{{\text{d}}_{2}} \end{array}\right. \end{array}$ (3)

$\begin{array}{c} \text{Moving }\;\;\;\;\;\text{ range }\;\;\;\;\;\text{ plot }\;\;\;\;\;\;\left\{ \begin{array}{c} \text{UCL}=\bar{X}+3\frac{\overset{―}{\text{MR}}}{{\text{d}}_{2}}\\ \text{CL}=0\\ \text{LCL}=\bar{X}-3\frac{\overset{―}{\text{MR}}}{{\text{d}}_{2}} \end{array}\right. \end{array}$ (4)

Where $\overset{―}{\text{MR}}$ represents the mean of the $\;\;\;\;\;\;\text{ M}{\text{R}}_{\text{i}}$ with $\text{M}{\text{R}}_{\text{i}}=\left|{\text{x}}_{\text{i}}-{\text{x}}_{\text{i}-1}\right|,{\text{x}}_{\text{i}}$ being the observation ${\text{d}}_{2}$ dan ${\text{D}}_{4}$ representing a constant.

The I-Chart monitors the average data over time, while the MR-Chart shows variation between successive measurements. Combined with the I-MR Chart, they provide a comprehensive view of process performance, especially when focusing on individual measurements over group averages. The I-Chart plots each individual data point at regular intervals to detect trends and changes in the process, capturing both common cause and special cause variations. Data in the I-Chart should be arranged chronologically for effective time-related performance analysis. The MR-Chart complements the I-Chart by displaying differences between successive data points. The I-MR control chart helps identify when a process moves out of statistical control and indicates the source of special cause variations [25].

### Steps of the analysis

The research methodology employed a sequential approach encompassing the following stages:

1. **Comprehensive Literature Review**: An in-depth exploration of existing literature was conducted to establish a theoretical framework and identify research gaps pertinent to the study objectives.
2. **Simulation Studies**: To evaluate the efficacy of the proposed approach under controlled conditions, simulation studies were implemented.
3. **Variable Definition and Data Collection**: Relevant variables were meticulously defined and operationalized, followed by the systematic collection of associated data.
4. **Data Partitioning**: Time series analysis was utilized to segment the collected data into distinct phases I and II, facilitating subsequent analysis.
5. **Autocorrelation Assessment**: The presence of autocorrelation within the data for each country was examined using autocorrelation function (ACF) and partial autocorrelation function (PACF) plots.
6. **Modeling**: In instances of significant autocorrelation, the XGBoost regression method was employed to obtain residual data and mitigate the autocorrelation effect.
7. **Statistical Process Control**: I-MR control charts were constructed based residual of XGBoost regression on to monitor process stability and identify potential anomalies.
8. **Conclusion and Recommendation Formulation**: Based on the findings derived from the preceding steps, comprehensive conclusions were drawn, and actionable recommendations were developed.

### Flowchart

**Fig 1:**
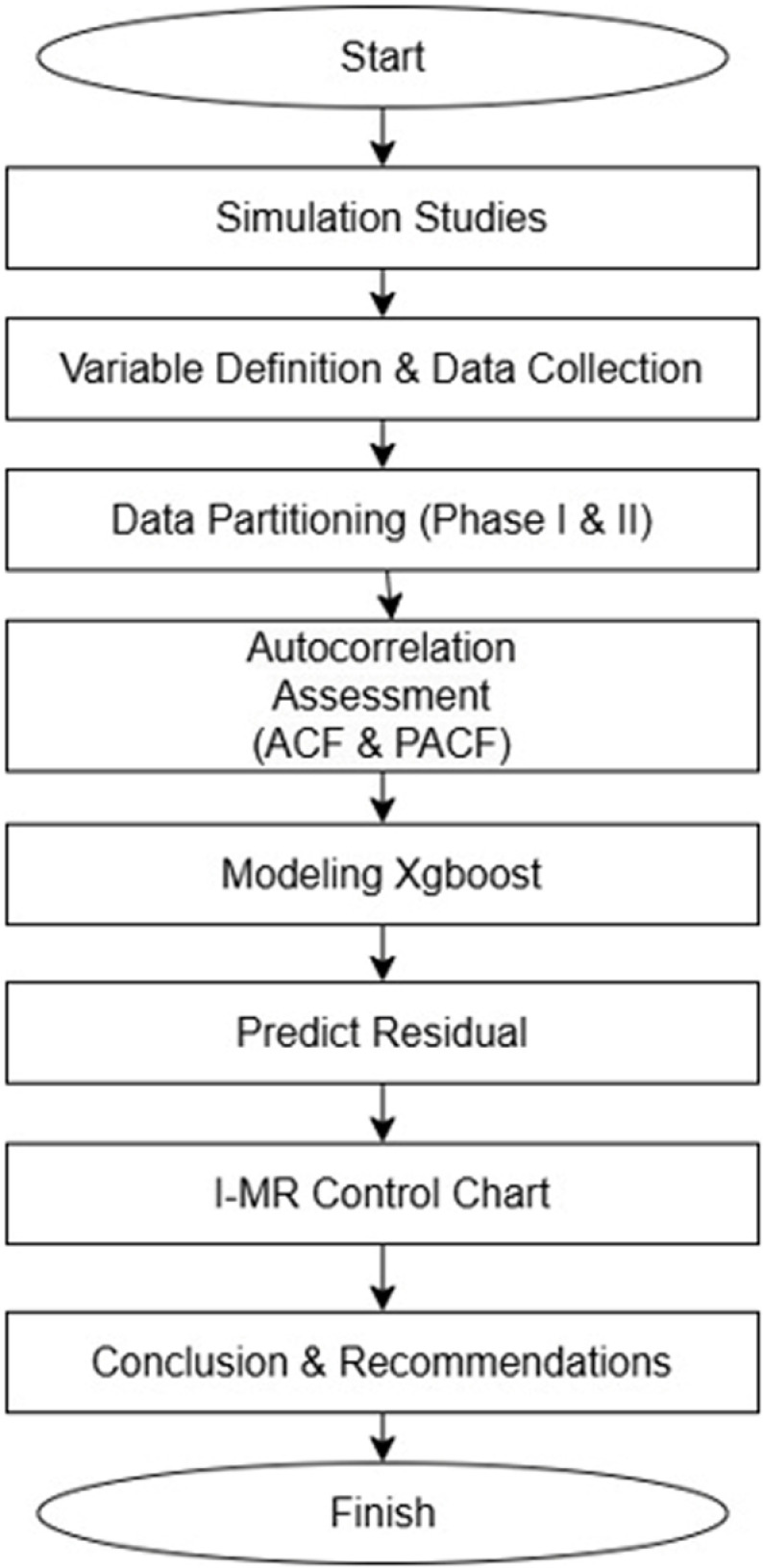
Flowchart.

This visual will help orient the reader to the framework of the study and offer a clearer perspective on how each section or aspect of the research interconnects. With this overview in mind, from Fig 1 flowchart illustrates the structured methodology used throughout this paper.

### Pseudocodes

By presenting this pseudocode, the reader can gain a more detailed understanding of how the algorithm operates, allowing for better comprehension of the computational approach or analysis applied throughout this study.

**Table:** 

Algorithm
**Input** : Training dataset *train_data*, Testing dataset *testing_data, XGBoost Model* **Output** : individual – moving range control chart value and control limit *individual_phaseI, individual_phaseII, MR_phaseI, MR_phaseII, I_CL,I_UCL, I_LCL, MR_CL, MR_UCL, MR_LCL* Function train_model(*train_data*) 1: set (*y*_*1*_) = shifted target column 2: set (*x*_*1*_) = lagged input column 3: combine *y*_*1*_ and *x*_*1*_ into *data* 4: *xgb_train* = XGBoost Dmatrix (*data*) 5: set (*max_depth*, *nrounds*) 6: Train model (*xgb_train*) 7: return(*xgb_model*) Function predict_residual(*xgb_model, train_data, test_data, d*_*2,*_ *D*) 8: predict(*xgb_train*) 9: *train_residuals* = residual(*xgb_train*) 10: *test_data* = XGBoost Dmatrix(*test_x*) 11: predict(*test_x*) 12: *test_residuals =* residual(*test_x*) 13: return(*train_residuals, test_residuals*) Funtion compute_control_chart(*train_residual*, *test_residual*) 14: for *i* in 1:length(*train_residuals*) do 15: *individual_phaseI*[*i*] = *train_residuals*[*i*] 16: *MR_phaseI*[*i+*1] = |*train_residuals*[*i+1*]-*train_residuals*[*i*]| 17: end for 18: for *j* in 1:length(*test_residuals)* do 19: *individual_phaseII*[*j*] = *train_residuals*[*j*] 20: *MR_phaseII*[*j+*1] = |*train_residuals*[*j+1*]-*train_residuals*[*j*]| 21: end for 22: *Avg_MR* = sum(*MR_phaseI*) / (length(*train_residuals*) - 1) 23: *constant =* 3 / *d*_*2*_ 24: *I_CL* = mean(*train_residuals*) 25: *I_UCL* = *MR_CL + constant × Avg_MR* 26: *I_LCL* = *MR_CL - constant × Avg_MR* 27: if *I_LCL < 0* then 28: *I_LCL* = 0 29: end if 30: *MR_CL* = *Avg_MR* 31: *MR_UCL = D × Avg_MR* 32: *MR_LCL* = 0 33: return(*individual_phaseI, individual_phaseII, MR_phaseI, MR_phaseII, I_CL,I_UCL, I_LCL, MR_CL, MR_UCL, MR_LCL*)

## Results and discussion

### Simulation Studies

The ARIMA model is a widely employed statistical method for time series forecasting, capable of capturing autocorrelation and non-stationarity inherent in such data. Prior to the application of more complex ensemble or machine learning models like XGBoost, extensive simulation studies utilizing ARIMA serve as a foundational step to characterize the underlying patterns and stochastic properties of time series [26]. “This study investigates the potential for enhancing predictive accuracy through the hybrid modeling of ARIMA and XGBoost algorithms. A simulation-based approach is employed to rigorously evaluate the model’s performance using synthetic datasets prior to real-world application. This methodology enables a comprehensive assessment of the model’s predictive capabilities under controlled conditions [27]. Three simulation scenarios were conducted, each generating 100 data points from AR(1), MA(1), and ARMA(1,1) processes. These datasets were subsequently partitioned into training (*n* = 70) and testing (*n* = 30) subsets for Phase I and Phase II analysis, respectively.

**Fig 2:**
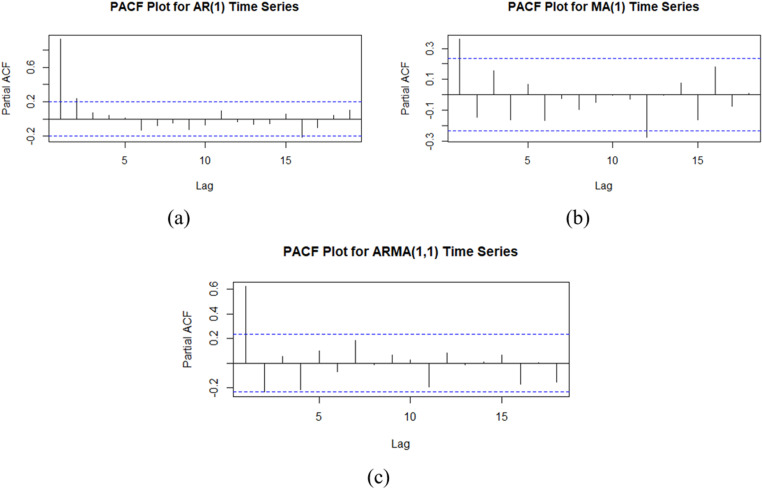
PACF Plot of Data Phase I: (a)AR (1), MA (1), and (c) ARMA (1,1).

Prior to extracting residual values via XGBoost regression, a lag structure analysis was conducted for each AR(1), MA(1), and ARMA(1,1) model in Phase I. PACF plots, as depicted in Fig 2, revealed a significant lag of one at the 0.05 level for all models. Subsequently, XGBoost models were constructed for each time series in Phase I, with their respective residuals serving as input for the subsequent Phase II modeling.

**Fig 3:**
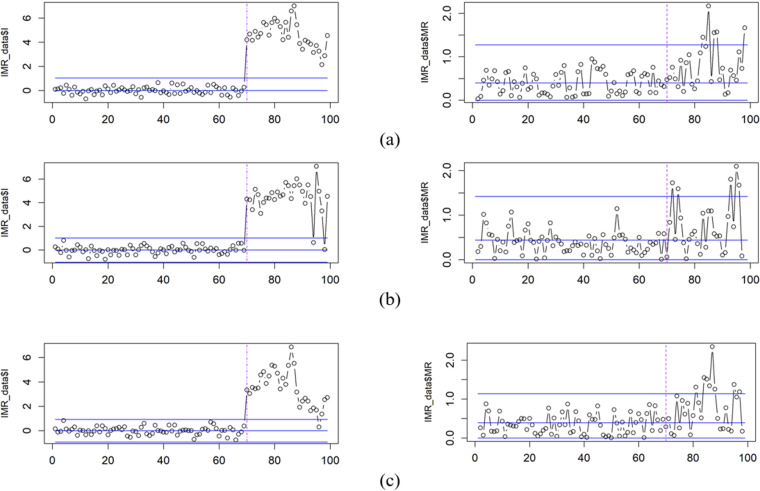
I-MR Control Charts for Data Phase I and Phase II: (a) AR (1), (b) MA (1), (c) ARMA (1,1).

Subsequent model diagnostics were conducted using I-MR control charts to assess the quality of model residuals. A two-phase approach was employed for control limit estimation and process monitoring. Phase I control limits were established based on initial residual data and subsequently used to monitor the stability of residuals in phase II. Fig 3 illustrates the I-MR control charts for the three models.

Table 1 presents a comparative analysis of out-of-control observations during Phase I and Phase II for three simulated models. Results indicate that the AR(1) model exhibited the highest frequency of out-of-control points in Phase I, while the ARMA(1,1) model demonstrated the greatest number of such occurrences in Phase II.

**Table 1:** Number of Out-of-Control Observations.

Scenario	OOC	
I	MR
AR (1)	30	5
MA (1)	29	6
ARMA (1,1)	29	8

### Xgboost Modelling for GDP Data

**Fig 4:**
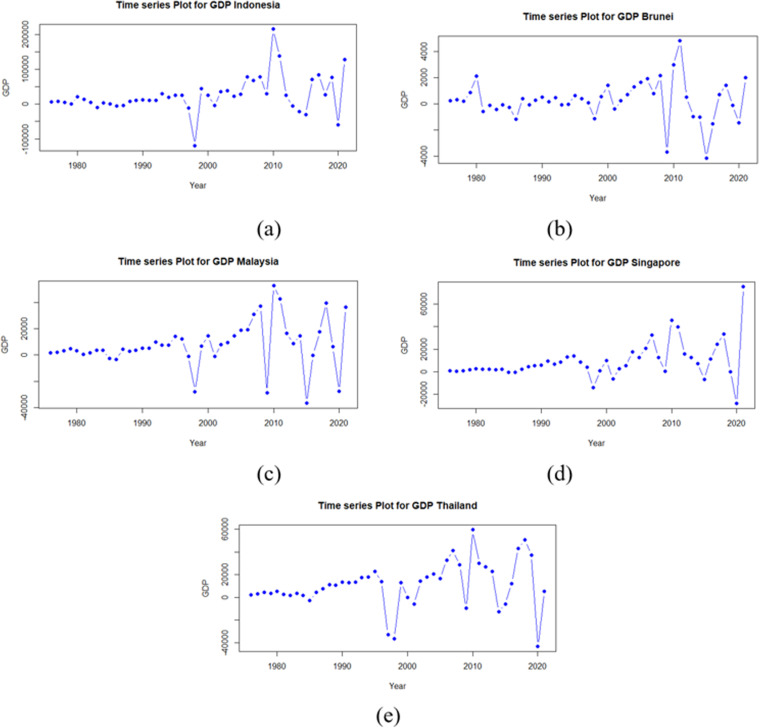
Time Series Plot of GDP Values: (a) Indonesia, (b) Brunei Darussalam, (c) Malaysia, (d) Singapore, (e) Thailand.

**Fig 5:**
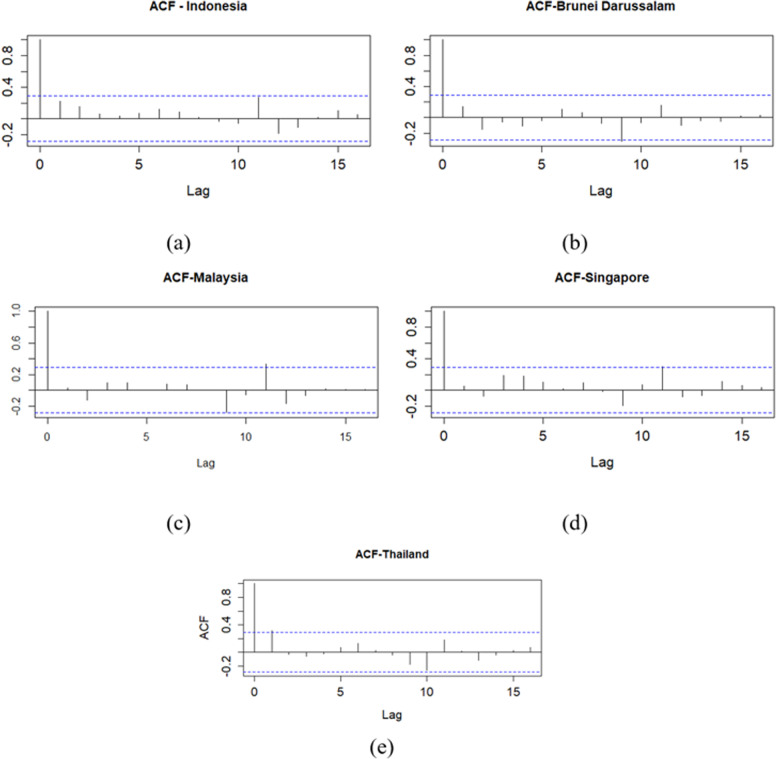
ACF plot for GDP Growth: (a) Indonesia, (b) Brunei Darussalam, (c) Malaysia, (d) Singapore, (e) Thailand.

**Fig 6:**
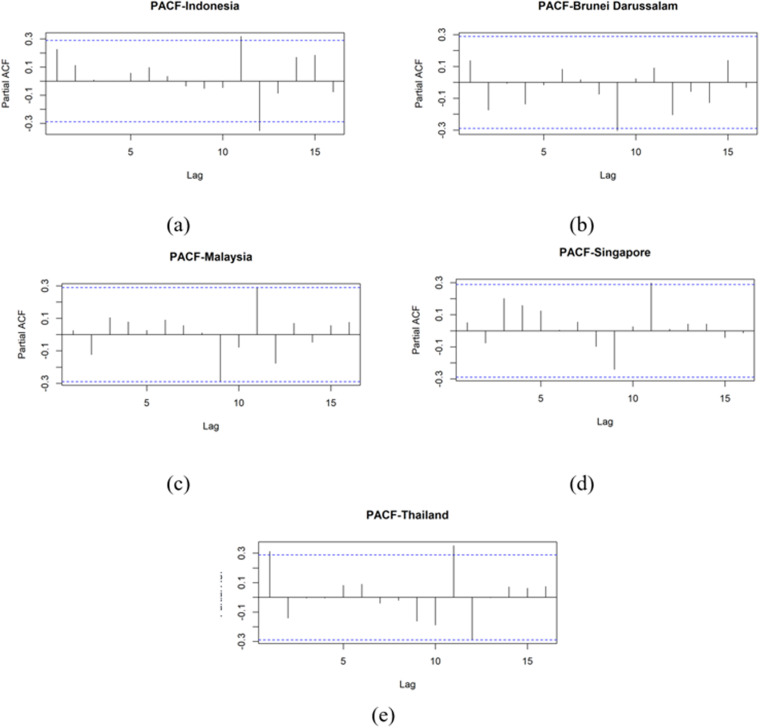
PACF plot for GDP Growth phase I: (a) Indonesia, (b) Brunei Darussalam, (c) Malaysia, (d) Singapore, (e) Thailand.

Time series plot of GDP in each country is presented in Fig 4. The partitioning of the dataset into training and testing subsets was guided by the observed patterns and fluctuations within the data. The training data employed in this study did not contain any instances of GDP values that deviated significantly from the overall distribution, either in terms of extreme lows or highs. Before time series forecasting model development, autocorrelation analysis was conducted. Fig 5 presents autocorrelation function (ACF) plots for GDP growth rates across the examined countries. The ACF plots exhibit significant autocorrelation in each case. Given the presence of autocorrelation, an XGBoost regression model was employed to forecast GDP growth. To inform feature engineering for the XGBoost model, PACF plots were analyzed. These plots, depicted in Fig 6, indicate the presence of significant autocorrelation at lag 1 for Thailand’s GDP at a 0.05 significance level. This finding suggests that incorporating the previous period’s GDP value as a feature in the XGBoost model may enhance predictive performance. The inputs for the XGBoost model in phase 1 are formed as y1(i-1), y2(i-1), y3(i-1), y4(i-1), and y5(i-1).

In Phase I, five GDP time series models were developed using XGBoost with a maximum depth of 3 and 50 boosting iterations (nrounds). The max depth of 3 prevents overfitting by limiting tree complexity, ensuring the model captures patterns without noise. Fifty iterations balance enough boosting rounds for convergence with minimal computational load. Table 2 highlights the model’s iterative improvement over 50 cycles, with decreasing mean absolute percentage error (MAPE) and root mean square (RMSE) values indicating enhanced predictive accuracy. Initially high errors drop significantly by the final iterations, reflecting optimized performance. This shows the model’s increasing reliability across different datasets.

**Table 2:** Evaluation Performance each Iteration.

Iteration	Indonesia		Brunei		Malaysia		Singapore		Thailand	
MAPE	RMSE	MAPE	RMSE	MAPE	RMSE	MAPE	RMSE	MAPE	RMSE
1	258.24	43,044	98.72	1,022	91.42	13,838	89.15	11,047	101.17	17,308
2	311.91	36,635	102.05	894	99.66	11,982	78.48	9,573	117.77	15,481
3	405.78	31,268	98.96	806	105.71	10,641	78.97	8,406	118.87	14,237
4	476.93	27,021	92.57	721	105.59	9,591	82.58	7,467	128.23	12,602
5	526.89	23,487	91.48	659	109.96	8,754	84.67	6,827	131.47	11,297
⋮	⋮	⋮	⋮	⋮	⋮	⋮	⋮	⋮	⋮	⋮
46	43.34	3,763	35.23	130	44.49	1,603	42.49	1,011	58.29	1,688
47	45.04	3,695	33.66	125	43.28	1,563	42.15	999	51.69	1,619
48	46.71	3,547	31.95	119	41.47	1,505	40.75	987	51.29	1,545
49	45.34	3,521	30.94	115	39.67	1,438	40.45	959	50.54	1,506
50	46.80	3,488	29.34	110	39.05	1,404	40.06	945	50.17	1,485

**Fig 7:**
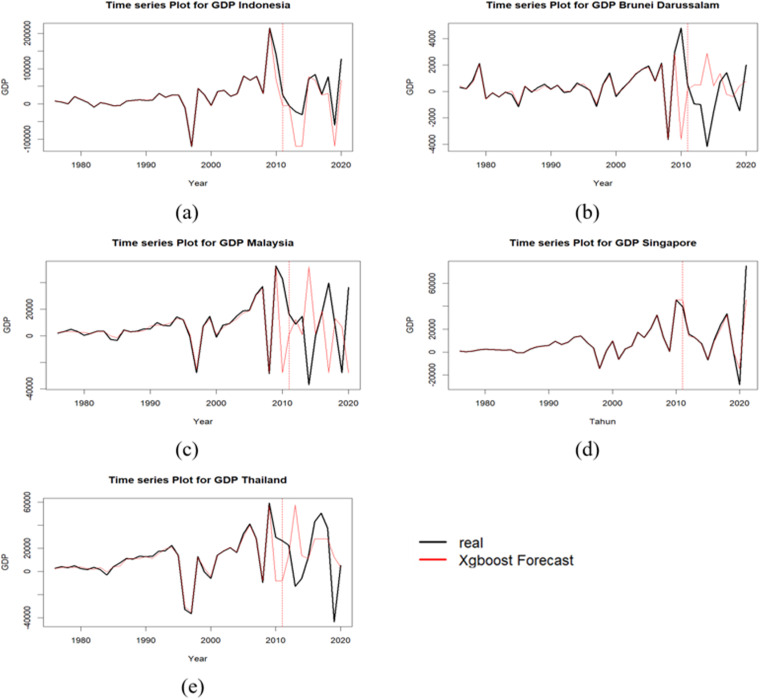
XGBoost Model for GDP Growth: (a) Indonesia, (b) Brunei Darussalam, (c) Malaysia, (d) Singapore, (e) Thailand.

As illustrated in Fig 7, the phase I predictions exhibited a strong alignment with the actual data patterns for all countries, as confirmed by the RMSE and MAPE values presented in Table 3. However, the phase II modeling revealed notable deviations between the predicted and actual GDP trajectories for Brunei, Malaysia, and Thailand. These discrepancies are likely attributable to structural shifts that occurred in the time series between the two phases. Despite these challenges, residual calculations were conducted for all countries in phase II to facilitate further analysis and evaluation.

**Table 3:** Model Evaluation Performance.

Country	MAPE	RMSE
Indonesia	46.80	3,488.53
Brunei	29.34	110.61
Malaysia	39.04	1,404.42
Singapore	40.06	945.94
Thailand	50.17	1,485.63

**Fig 8:**
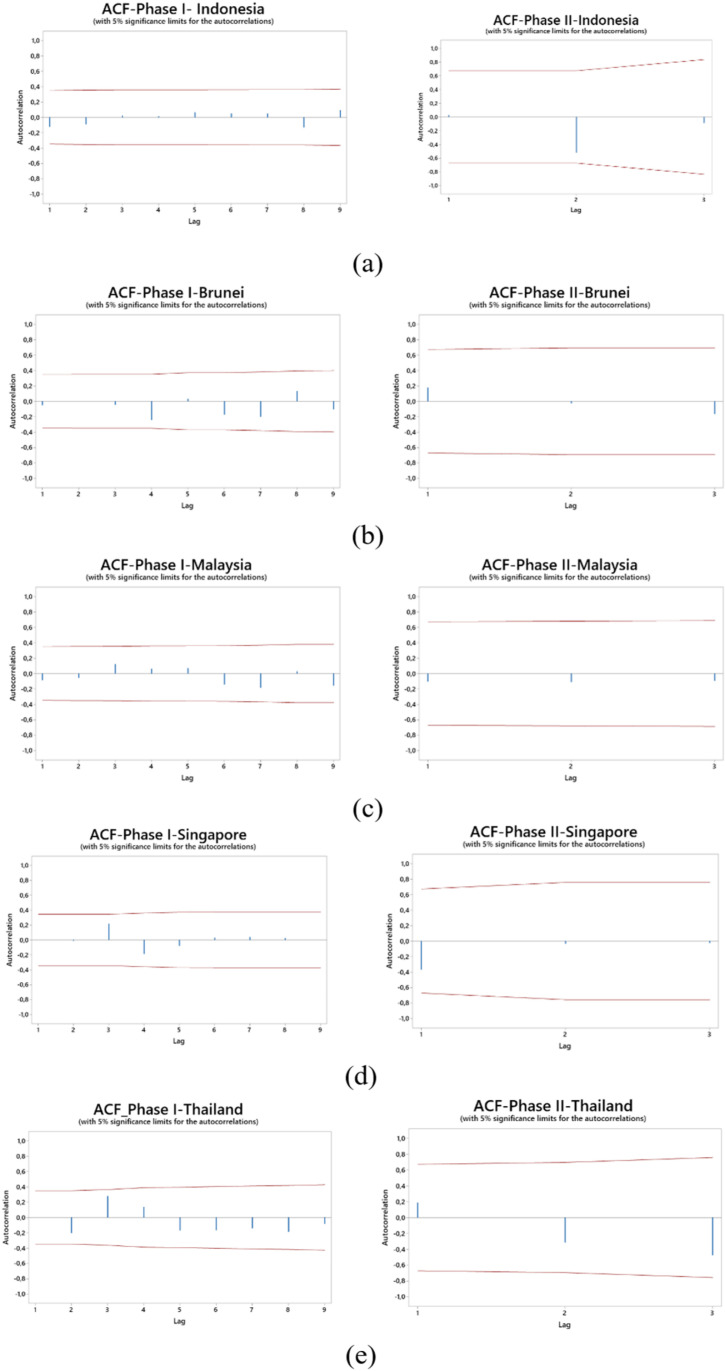
ACF residual plot phase I and II: (a) Indonesia, (b) Brunei Darussalam, (c) Malaysia, (d) Singapore, (e) Thailand.

To assess the adequacy of the XGBoost model for subsequent control chart monitoring, residual diagnostics were conducted. Specifically, the ACF of the residuals was examined to verify the absence of autocorrelation, a critical assumption for control chart effectiveness. As depicted in Fig 8, the ACF plot reveals no significant autocorrelation at any lag, indicating that the XGBoost model effectively captures the underlying data structure and generates residuals consistent with the white noise assumption.

### Monitoring Residual Xgboost using I-MR Control Chart

**Fig 9:**
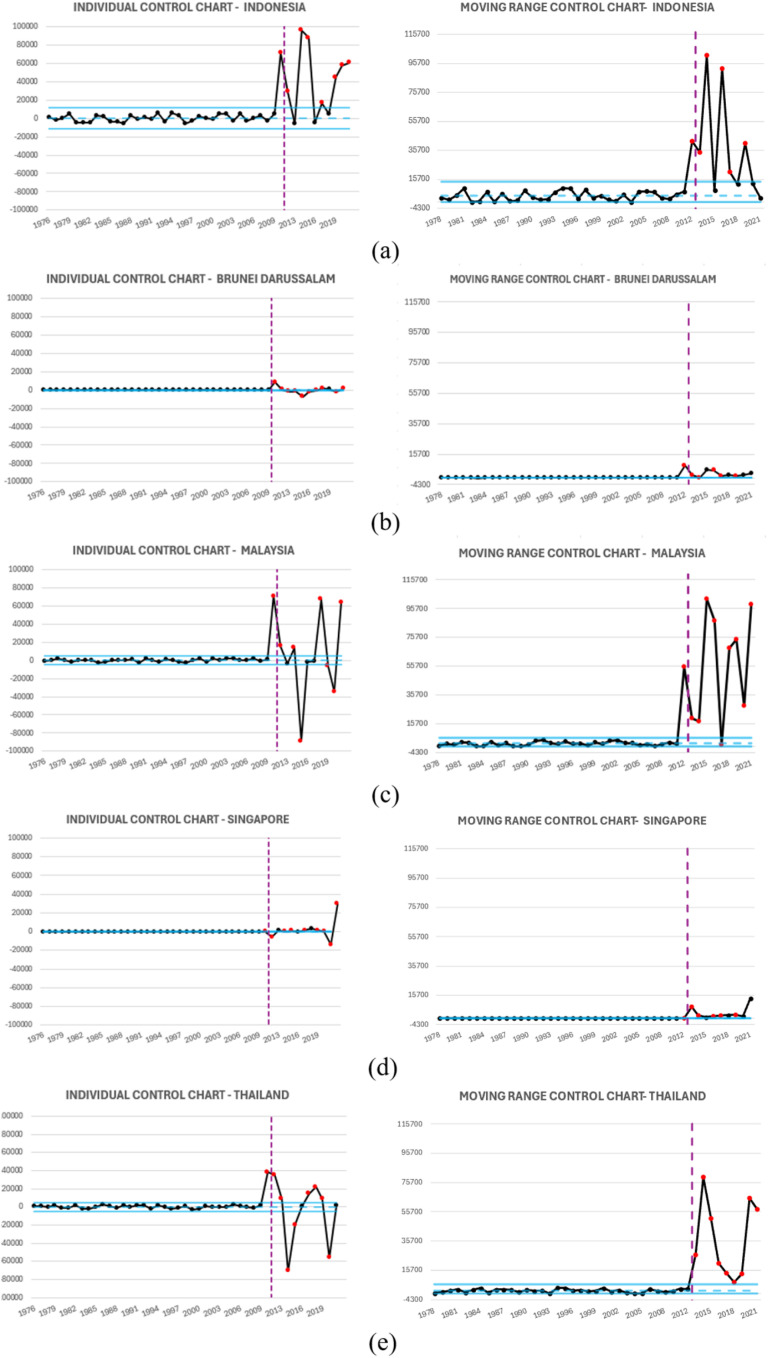
I-MR Residual Control Chart: (a) Indonesia, (b) Brunei Darussalam, (c) Malaysia, (d) Singapore, (e) Thailand.

The I-MR control chart was employed to assess the statistical control of residuals generated from the modeling process which presents in Fig 9. A two-phase approach was adopted. In Phase I, control limits were established based on the initial residual data. The GDP values corresponding to this phase exhibited statistical control, as evidenced by the absence of data points exceeding the control limits. Consequently, the analysis progressed to Phase II.

During Phase II, the I-MR control chart, incorporating distinct control limits for this phase, revealed that residual values for all countries exceeded the established thresholds at certain points. These findings indicate the presence of significant and anomalous fluctuations in GDP values.

### Comparison with Time Series Modeling using ARIMA

To further evaluate the effectiveness of different forecasting approaches, this study compares traditional forecasting using ARIMA with our proposed model, XGBoost models, in terms of their predictive performance and suitability for residual-based I-MR control charts. ARIMA, a widely used time series forecasting method, requires careful parameter tuning, typically based on ACF and PACF analysis. To manage this, we used Auto ARIMA. This can automates the selection of the optimal parameters by minimizing the Akaike Information Criterion (AIC) or Bayesian Information Criterion (BIC). Auto ARIMA reduces manual intervention and optimizes the model selection.

**Fig 10:**
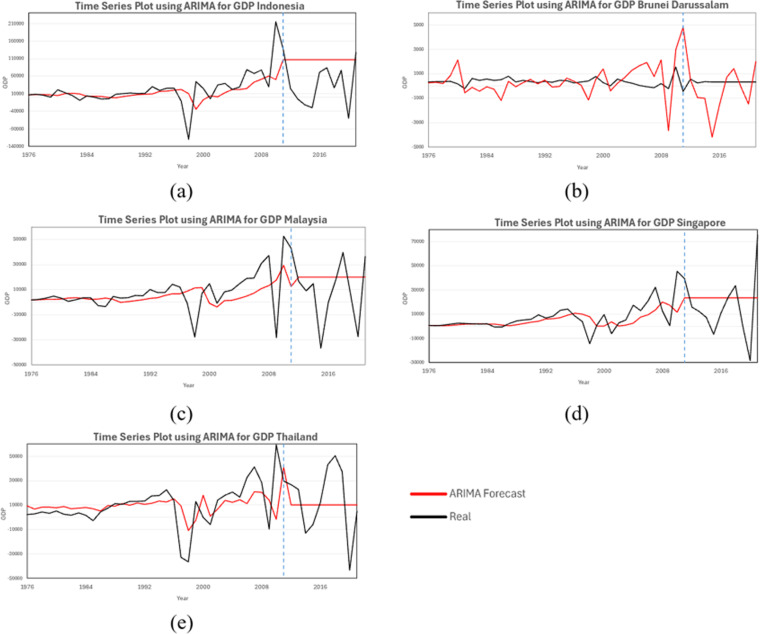
ARIMA Forecast plot for GDP Growth: (a) Indonesia, (b) Brunei Darussalam, (c) Malaysia, (d) Singapore, (e) Thailand.

Comparing with the evaluation performance from XGBoost model in Table 4, showed that the RMSE and MAPE values for ARIMA models across different countries were consistently higher than those of XGBoost. This suggests that ARIMA struggles to capture the underlying patterns in GDP growth data, leading to significant forecast deviations. Auto ARIMA, while optimizing ARIMA parameters automatically, did not substantially improve forecast accuracy and remained less reliable than XGBoost. From Fig 10, show noticeable deviations from actual GDP growth trends, particularly in Phase II, where structural shifts and economic fluctuations appear more pronounced. The forecasts generated by ARIMA tend to lag behind actual values, and in some cases, overestimate or underestimate GDP growth trends. In contrast, XGBoost models align more closely with the observed data, effectively capturing both short-term and long-term fluctuations.

**Table 4:** Model Evaluation Performance Comparison.

Country	XGBoost		Auto ARIMA	
MAPE	RMSE	MAPE	RMSE
Indonesia	46.80	3,488.53	127.35	2,383.57
Brunei	29.34	110.61	1,920.66	26,692.84
Malaysia	39.04	1,404.42	6,824.35	26,683.95
Singapore	40.06	945.94	97.06	26,459.40
Thailand	50.17	1,485.63	383.14	91,981.23

**Fig 11:**
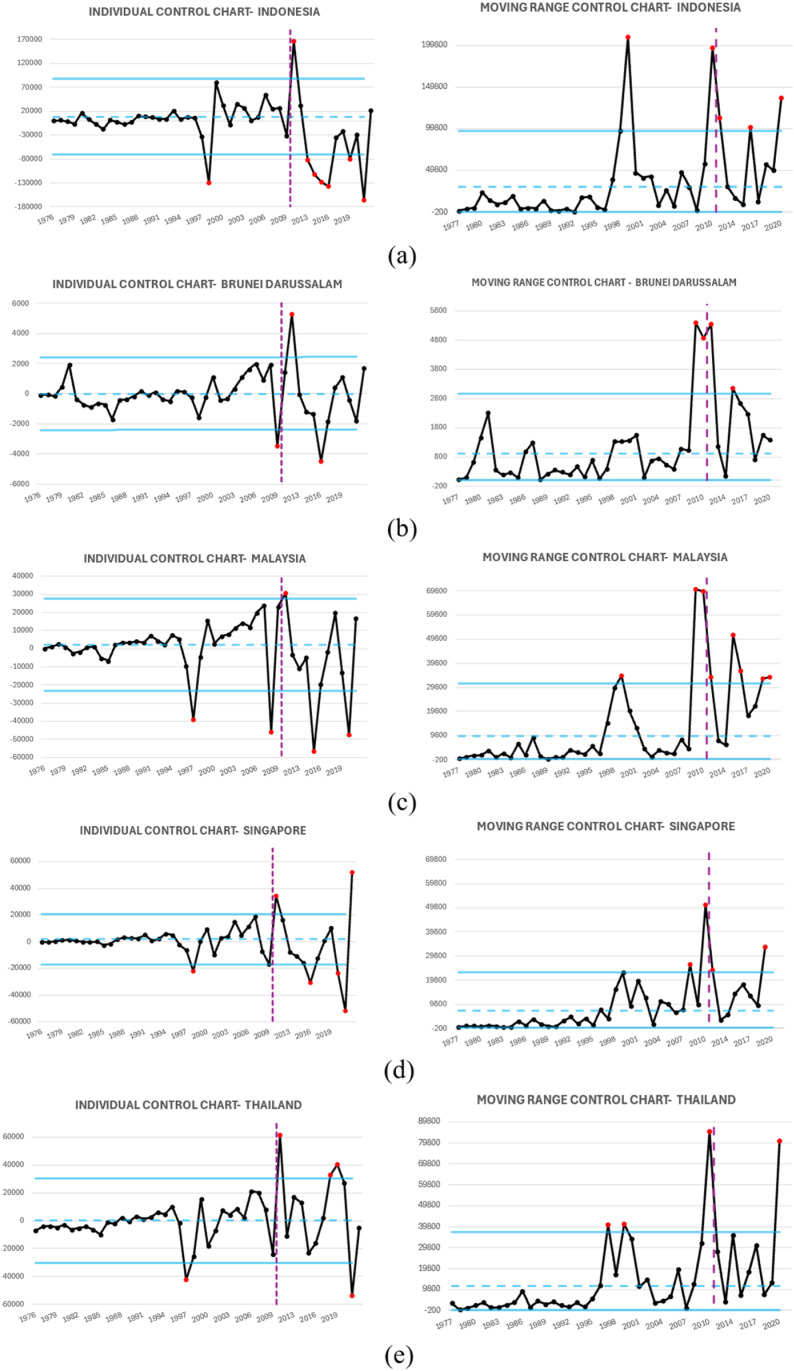
I-MR ARIMA Residual based Control Chart: (a) Indonesia, (b) Brunei Darussalam, (c) Malaysia, (d) Singapore, (e) Thailand.

The I-MR control charts in Fig 9 (XGBoost residual-based) and Fig 11 (ARIMA residual-based), along with Table 5, show that XGBoost detects more out-of-control (OOC) points in the I chart compared to ARIMA. This indicates that XGBoost is more sensitive to structural shifts in GDP trends, particularly in Brunei and Singapore, where it identifies more deviations. In contrast, ARIMA’s lower OOC count suggests it smooths out variations excessively, potentially missing key economic changes. The MR chart results further support XGBoost’s stability, as its residuals exhibit more controlled variability, especially in Malaysia, while ARIMA residuals show greater fluctuations.

**Table 5:** Comparison of Out-of-Control Counts Across Models.

Country	XGBoost		ARIMA	
I	MR	I	MR
Indonesia	8	6	9	6
Brunei	10	9	4	6
Malaysia	8	9	5	9
Singapore	11	10	6	6
Thailand	9	9	6	5

Although XGBoost has a higher OOC count, this does not indicate instability but rather a better ability to capture GDP anomalies. Table 5 confirms that XGBoost identifies real economic shifts, while ARIMA’s lower OOC count may indicate an inability to detect significant changes. Additionally, XGBoost’s MR chart remains more stable across countries, reinforcing its robustness. Given GDP’s nonlinear nature, XGBoost’s adaptability makes it the superior model for forecasting and monitoring, offering better anomaly detection and more reliable insights than ARIMA.

## Discussion and limitation

This study proposes a hybrid modeling framework to enhance GDP forecasting accuracy for Malaysia, Brunei, Singapore, Thailand, and Indonesia. The methodology incorporates time series models - AR(1), MA(1), and ARIMA(1,1) - as foundational components. To capture potential non-linear relationships and improve predictive power, these time series models are subsequently integrated with the ensemble learning technique, XGBoost. The resulting forecasts are subjected to rigorous monitoring using I-MR control charts to assess the stability and reliability of the model’s performance over time.

It is essential to acknowledge the limitations inherent in this approach. The effectiveness of the hybrid model is contingent upon the underlying assumption that the GDP data for the target countries exhibit characteristics amenable to the specified time series models. Deviations from these assumptions could potentially compromise the accuracy of the forecasts. Moreover, the successful application of XGBoost is predicated on meticulous hyperparameter tuning to prevent overfitting and optimize model performance. The quality and quantity of the training data are also critical factors influencing the algorithm’s predictive capabilities. The efficacy of I-MR control charts in monitoring residual values is reliant on the assumption of stable and normally distributed error terms. Violations of these assumptions may lead to inaccurate assessments of model performance.

For modelling comparison, ARIMA models rely on strong assumptions of linearity and stationarity, which may limit their ability to model complex GDP fluctuations. In contrast, XGBoost introduces a more flexible, non-linear approach, enabling it to learn from intricate economic patterns, adapt to structural changes, and provide more accurate predictions. By incorporating I-MR control charts, both models’ residuals are monitored to evaluate their stability, revealing that XGBoost outperforms ARIMA in detecting anomalies and economic shifts.

Despite its advantages, XGBoost is not without limitations. While it successfully captures non-linear relationships, its effectiveness depends on meticulous hyperparameter tuning to prevent overfitting and ensure generalizability. In contrast, ARIMA models require parameter estimation but follow a more structured selection process (e.g., ACF and PACF analysis), making them more interpretable. Moreover, the I-MR control chart assumes that residuals follow a stable and normally distributed pattern, which may not always hold for either model.

The identification of anomalies or out-of-control signals within GDP growth trajectories can offer invaluable insights for policymakers and economists. These aberrant signals often serve as indicators of latent economic challenges, including inflation, unemployment, or disruptions in supply chains. By proactively identifying these anomalies, policymakers can implement well-targeted interventions, such as adjusting interest rates, modifying fiscal policies, or introducing stimulus packages, to effectively stabilize the economy.

Furthermore, the early detection of anomalies can enable policymakers to anticipate and mitigate potential economic crises. By understanding the underlying causes of these anomalies, policymakers can take proactive steps to address the root issues and prevent their escalation. For instance, if an anomaly is indicative of inflationary pressures, policymakers can implement measures to curb inflation, such as raising interest rates or reducing government spending.

Additionally, economists can leverage the information derived from anomaly detection to refine their economic forecasting models. By incorporating these insights into their models, economists can improve the accuracy and reliability of their predictions, providing policymakers with more informed guidance for decision-making. Moreover, the identification of anomalies can help economists identify emerging economic trends and patterns, enabling them to anticipate future developments and advise on long-term strategic planning.

## Conclusion

This research investigates the application of I-MR control charts to monitor and potentially regulate the GDP growth trajectories of five G20 nations. A novel approach is proposed, employing the residuals derived from XGBoost regression as input for the control charting process. This methodology addresses the autocorrelation challenge inherent in traditional time series models, as evidenced in previous studies. By incorporating XGBoost, the study develops a more robust predictive framework for GDP forecasting. While Phase I predictions demonstrate adequate alignment with actual data, Phase II models exhibit deviations, suggesting avenues for refinement. The analysis underscores the efficacy of I-MR control charts based on XGBoost residuals in monitoring GDP forecast accuracy. Phase I residuals conform to control limits, whereas Phase II reveals out-of-control signals, indicative of significant GDP fluctuations. This research contributes to enhancing economic stability monitoring for the selected G20 countries. Ongoing refinement of the XGBoost model through hyperparameter optimization is essential for improving predictive accuracy. Future research could explore the integration of more sophisticated machine learning algorithms or hybrid models to further enhance GDP forecasting capabilities.

## Supporting information

S1 Data (XLSX)
